# Health-related quality of life (QoL) in patients with advanced melanoma receiving immunotherapies in real-world clinical practice settings

**DOI:** 10.1007/s11136-020-02520-7

**Published:** 2020-05-13

**Authors:** Richard W. Joseph, Frank Xiaoqing Liu, Alicia C. Shillington, Cynthia P. Macahilig, Scott J. Diede, Vaidehi Dave, Qing Harshaw, Todd L. Saretsky, Alan Simon Pickard

**Affiliations:** 1 Department of Oncology (Medical), Mayo Clinic, Jacksonville, FL USA; 2 Merck & Co., Philadelphia, PA USA; 3 EPI-Q, Inc., 915 Harger Rd, Suite 350, Oak Brook, IL 60523 USA; 4 Medical Data Analytics (MDA), Parsippany, NJ USA; 5 Merck & Co., North Wales, PA USA; 6 Pharmacy Systems, Outcomes and Policy, College of Pharmacy, 833 South Wood Street, Chicago, IL 60612 USA

**Keywords:** Melanoma, Metastatic, Immunotherapy, Quality of life, Patient reported outcomes

## Abstract

**Background:**

Pembrolizumab (PEMBRO) and ipilimumab + nivolumab (IPI + NIVO) are approved advanced melanoma (AM) immunotherapies. To address limited health-related quality of life (QoL) real-world evidence with immunotherapies in AM, we compared QoL in AM patients receiving either treatment in clinical practice.

**Methods:**

A prospective US observational study enrolled adult AM patients initiating first-line PEMBRO or IPI + NIVO between June 2017 and March 2018. Endpoints included the QLQ-C30 global health score (GHS) and EuroQol visual analog scale (EQ-VAS) scores. Mean changes were compared using repeated measures mixed-effects models and are presented covariate adjusted.

**Results:**

225 PEMBRO and 187 IPI + NIVO patients were enrolled. From baseline through week 24, PEMBRO was associated with 3.2 mean GHS score increase (95% CI 0.5, 5.9; *p* = .02), while no change was observed with IPI + NIVO; 0.2 (95% CI − 2.6, 3.0; *p* = 0.87). Among objective treatment-responders, GHS scores associated with PEMBRO increased 6.0 (95% CI 3.1, 8.8; *p* < .0001); IPI + NIVO patients increased 3.8 (95% CI 0.8, 6.9; *p* = .01). In treatment non-responders, IPI + NIVO was associated with GHS/QoL deterioration of − 3.7 (95% CI − 6.8, − 0.6; *p* = .02), PEMBRO non-responders demonstrated no change; 0.7 (95% CI − 2.3, 3.7; *p* = 0.6). Between treatments, PEMBRO patients increased 2.6 greater in EQ-VAS (95% CI 0.6, 4.5; *p* = .01) vs IPI + NIVO at 24 weeks.

**Conclusions:**

PEMBRO was associated with better 24-week QoL compared to IPI + NIVO in actual clinical practice settings. Real-world data has known limitations, but with further confirmation these results may have implications for treatment selection.

**Electronic supplementary material:**

The online version of this article (10.1007/s11136-020-02520-7) contains supplementary material, which is available to authorized users.

## Background

Melanoma is a disease associated with clinically significant levels of distress at the time of diagnosis and treatment initiation, decreasing over time [1–3]. However very little is known about the impact of advanced melanoma (AM) on quality of life (QoL) outside the clinical trial setting. Patient-reported outcomes (PROs) specifically those that focus on health-related QoL, are used in melanoma clinical trials to supplement clinical outcomes [4, 5]. Assessing PROs in oncology is important as adverse events (AEs) and cancer symptoms affect subjective experience and functioning [6]. In melanoma trials, PROs are generally collected during therapy and for an abbreviated period after patients conclude treatment with investigational agents [4, 5]. This provides limited insight into real-world therapy effectiveness in patients more heterogeneous than are included in clinical trials.

Most evidence to support the use of immunotherapies derives from trials. Pembrolizumab (PEMBRO) and ipilimumab + nivolumab (IPI + NIVO) are approved immunotherapies for AM [7, 8]. In KEYNOTE-006, PEMBRO demonstrated prolonged progression-free survival (PFS) and overall survival (OS) compared to ipilimumab (IPI) [9]. This study also showed patients treated with PEMBRO experienced less QoL deterioration at week 12 compared to IPI. Subjects receiving IPI experienced more grade 3–5 AE, (59% IPI vs 17% PEMBRO), which may impact QoL [5, 9]. In CheckMate-067, which examined patients with AM treated with nivolumab (NIVO) or IPI + NIVO, and in CHECKMATE-069, which included patients treated with either IPI or IPI + NIVO, no treatments were associated with clinically meaningful change in QoL when measured by The European Organisation for Research and Treatment of Cancer (EORTC) Quality of Life Questionnaire C30 (QLQ-C30) or the EQ-5D [4, 10]. There are no studies reporting QoL in real-world settings in patients who receive PEMBRO or IPI + NIVO. Thus, the aim of this study was to examine QoL outcomes associated with PEMBRO treatment compared to IPI + NIVO when administered as first-line therapy in AM patients.

## Methods

### Study design

We report 24-week data from a cohort study of consecutively enrolled AM patients initiating PEMBRO or IPI + NIVO as first line in 9 US-based academic and satellite centers. Sites were identified in national provider databases and recruited by telephone. To be eligible, academic centers needed to treat ≥ 15 AM patients yearly and satellite centers needed to treat ≥ 7. Sites must have indicated use of both PEMBRO and IPI + NIVO in an attempt to balance enrollment between treatments within sites. Sites were blinded to sponsor and vice versa to avoid impact on treatment selection. Patients with histologically-confirmed unresectable Stage III or metastatic / Stage IV melanoma (excluding uveal or ocular melanoma), age ≥ 18, initiating first-line PEMBRO or IPI + NIVO between June 2017 and March 2018 were consented and enrolled. Patients were mailed paper PRO questionnaires within 10 days of starting therapy and weeks 6, 12, 18, 24 following therapy initiation. Patients were given a 10-day window before and after PRO assessment timepoints to complete questionnaires and were sent reminders by mail if not received. PRO collection continued after treatment discontinuation through death, most recent site visit date or loss to follow-up. This study was approved by the New England Institutional Review Board (Approval #120161001).

### Data collection

Sites collected baseline clinical data via chart review. Chart review data consisted of demographics (e.g., age, American Joint Committee on Cancer [AJCC] version 7 clinical stage, Eastern Cooperative Oncology Group [ECOG] performance status, lactate dehydrogenase [LDH], Charlson Comorbidity Index [CCI], and presence of an autoimmune comorbidity.^1^ Clinician-assessed best objective response to therapy was collected, with complete and partial response recorded as ‘therapy objective response’.

PROs were assessed using the EORTC-QLQ-C30 [11] and EQ-5D-5L [12], both of which are commonly used in melanoma clinical trial settings [3, 4, 10]. The EORTC QLQ-C30 includes 30 items, including a two-item global health status (GHS)/QoL scale rated from 1 to 7 with 1 indicating “very poor” health and QoL and 7 indicating “excellent” health and QoL in the past week. Five dimensions similarly scaled rated physical, role, emotional, cognitive, and social functioning rated from “very poor" to “excellent”. Symptoms were assessed including fatigue, nausea/vomiting, pain, dyspnea, sleep disturbance, appetite loss, constipation, diarrhea, and financial impact rated on a scale from 1 to 4, with 1 meaning “no impact” and 4 meaning “very much”. The EQ-5D-5L includes a health state classifier system and visual analog scale (EQ-VAS), where patients rate general state of health at the current date from 0 to 100 with 0 representing worst imaginable health, and 100 representing best. The present analysis focused on the overall rating of health as represented by the QLQ-C30 GHS/QoL score and the EQ-VAS.

### Statistical analyses

QoL analyses were exploratory in the study protocol and followed a pre-specified analysis plan. The analytic cohort included all enrolled patients completing at least one PRO instrument. An instrument was considered complete according to the missing item guidance in the EORTC QLQ-C30 and EQ-5D-5L manuals [11, 12].

PRO compliance was defined as the proportion of patients completing the instrument among those expected to complete at each time point, excluding deaths from the denominator. PRO completion was defined as the proportion of patients completing the instrument among all enrolled subjects, including deaths in the denominator.

Differences in patient characteristics were assessed using *t* tests for continuous data, and *χ*^2^ tests for categorical data. The EORTC QLQ-C30 GHS/QoL score was calculated and standardized (range 0–100) [12]. Analyses were performed through week 24 using repeated measures mixed models. Least squares (LS) mean changes from baseline at each time period were calculated adjusting for age, ECOG performance states, baseline PRO score, AJCC stage, therapy objective response and presence of comorbidity. Analysis assessed the LS mean score change from baseline within treatments and between treatments at each timepoint using *F* tests. In addition to a week by treatment interaction term in the model to assess change at each time period, treatment by therapy objective response interaction was used in the same model to examine the impact of response within each treatment over time. We examined the proportion of patients who improved, remained stable or deteriorated at each week compared to the previous measurement period via a X^2^ statistic, with clinically meaningful differences defined by a 10-point change from baseline for EORTC QLQ-C30 scales [13] and a 7-point change for the EQ-VAS [14]. We controlled for factors specified in the a priori analysis plan and potential confounders unbalanced between groups as identified in univariate analysis. Included in initial models were the covariates ECOG performance status, BRAF mutation vs wild type, PD-L1 expression positive vs negative, (each specified a priori) LDH status (normal, elevated 1–2 times upper limit of normal [ULN] or > 2 times ULN), and comorbidity. To avoid overfitting, parameters were entered into the model then removed. Covariates retained in regression models were those *p* < 0.1 (baseline QLQ-C30 GHS, comorbidity index, ECOG at baseline, age, AJCC stage, autoimmune comorbidity). As Qol, and not specific symptom scores in the EORTC QLQ-C30 were primary outcome of this analysis, symptom score changes from baseline were analyzed only descriptively.

## Results

### Patients

Four hundred and twelve patients were enrolled, 225 receiving PEMBRO, and 187 receiving IPI + NIVO. Overall, patients were mostly male (57%), Caucasian (93%), mean (SD) age of 63 (8.7) years, with an ECOG performance status of 0 or 1 (77%) at advanced diagnosis. Patients treated with PEMBRO were significantly older than those treated with IPI + NIVO, with 49% and 37% being > age 65, respectively (*p* = 0.01). Patients treated with PEMBRO had higher mean (SD) comorbidity index of 1.0 (1.3) compared with 0.6 (0.9) with IPI + NIVO (*p* < 0.001), and significantly more autoimmune comorbidity of 11% vs 4%, respectively (*p* = 0.01). Patients treated with PEMBRO were more often AJCC Stage IV compared to IPI + NIVO, 93% vs 85% (*p* = 0.02) (Table 1). There was no difference in objective response rates to therapy—PEMBRO 62% versus IPI + NIVO 64% (*p* = 0.8).

**Table 1:** Baseline characteristics

	PEMBRO *N* = 225	IPI + NIVO *N* = 187	*p* value
Gender, *n* (%)			
Female	104 (46.2)	74 (39.6)	.18
Male	121 (53.8)	113 (60.4)	
Race, *n* (%)			
Caucasian	205 (91.1)	177 (94.7)	.66
African American	4 (1.8)	2 (1.1)	
American Indian/Alaskan Native	7 (3.1)	3 (1.6)	
Asian	5 (2.2)	4 (2.1)	
Other	4 (1.8)	0 (0.0)	
Ethnicity, Hispanic/Latino (%)	13 (5.8)	4 (2.1)	
Age at therapy initiation (years)			
Mean (SD)	63.8 (8.9)	61.5 (8.3)	.006
Age at therapy initiation by 65-Year age group, *n* (%)			
< 65	114 (50.7)	118 (63.1)	.01
> 65	111 (49.3)	69 (36.9)	
Charlson Comorbidity Index Mean (SD)	1.0 (1.3)	0.6 (0.9)	.000
Autoimmune comorbidity	25 (11.1)	8 (4.3)	.01
AJCC stage—v7, n (%)			
IIIB/IIIC	17 (7.6)	28 (15.0)	.02
IV	208 (92.4)	159 (85.0)	
Metastasis stage			.24
m1a	89 (42.8)	74 (46.5)	
m1b	19 (9.1)	21 (13.2)	
m1c	100 (48.1)	64 (40.3)	
Metastases sites at advanced melanoma diagnosis, *n* (%)			
Lymph nodes	118 (52.4)	117 (62.6)	.04
Distant skin	86 (38.2)	48 (25.7)	.007
Lung	68 (30.2)	49 (26.2)	.37
Bone	39 (17.3)	25 (13.4)	.27
Liver	27 (12.0)	20 (10.7)	.68
Brain	19 (8.4)	8 (4.3)	.09
ECOG score at advanced diagnosis, *n* (%)			
0–1	179 (79.6)	137 (73.3)	.13
2–4	46 (20.4)	50 (26.7)	
LDH, *n* (%)			
Normal	146 (64.9)	138 (73.8)	.10
1–2 ULN	58 (25.8)	32 (17.1)	
> 2-time ULN	21 (9.3)	17 (9.1)	
Baseline QLQ-C30 GHS/QoL	58.8 (22.3)	59.5 (22.6)	.75
Baseline EQ-VAS, mean (SD)	65.0 (21.4)	67.7 (21.2)	.21

*ECOG* Eastern Cooperative Oncology Group, *LDH* lactate dehydrogenase, *SD* standard deviation, *ULN *upper limits of normal, *PEMBRO* pembrolizumab, *IPI + NIVO* Ipilimumab + NIVOLUMAB

### PRO questionnaires completion and compliance rates

Completion rates for both instruments at each timepoint within treatment groups were identical. At week 24 the completion rates were 95% and 96%, respectively for PEMBRO and IPI + NIVO. By week 6, three patients in the PEMBRO arm were lost to follow-up. At week 24, compliance rates were 99% and 100% for PEMBRO and IPI + NIVO, respectively. Missing data was were rare (< 2% total instruments) and no individual items were missed within instruments. (Supplemental Table 1).

### EORTC QLQ-C30 GHS/QoL LS Mean change from baseline

**Fig. 1:**
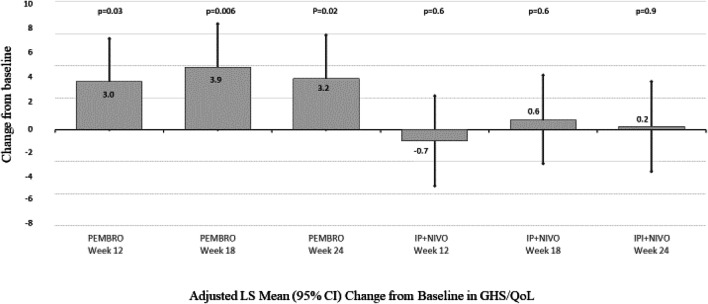
Adjusted LS Mean Change from Baseline in QLQ-C30 GHS. Repeated measures mixed model with treatment by objective treatment response interaction, treatment by week interaction controlling for baseline QLQ-C30 GHS, comorbidity index, ECOG at baseline, age, AJCC stage, autoimmune comorbidity, therapy objective response to therapy. Positive number represents improvement in score, negative number represents deterioration. *GHS/QoL* global health quality of life scale, *LS* least squares, *PEMBRO* pembrolizumab, *IPI + NIVO* ipilimumab and nivolumab, *CI* confidence interval

Within-treatment adjusted LS mean scores for PEMBRO increased significantly, indicating improved QoL from baseline in patients at week 12 (Fig. 1). By week 24, PEMBRO patients reported a statistically significant 3.2-point increase (95% CI 0.5, 5.9; *p* = 0.02). No significant changes from baseline were noted within the IPI + NIVO treatment arm, 0.2-point change (95%CI − 2.6, 3.0; *p* = 0.9). (Fig. 1).

**Fig. 2:**
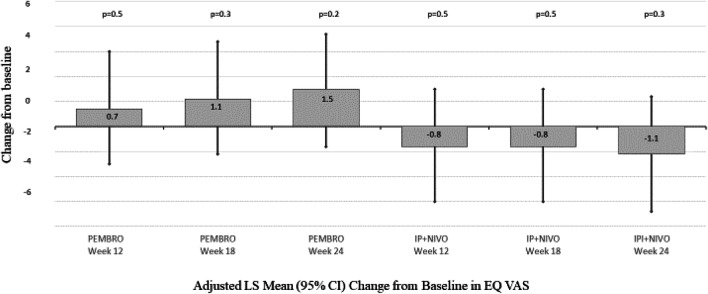
Adjusted LS Mean (95% CI) Change from Baseline in EQ-VAS. Repeated measures mixed model with cohort by objective response interaction, cohort by week interaction controlling for baseline EQ-VAS comorbidity index, ECOG at baseline, age, AJCC stage, autoimmune comorbidity, therapy objective response to therapy. *AJCC* American Joint Committee on Cancer, *ECOG* Eastern Cooperative Oncology Group, *CI* confidence interval, *GHS/QoL* global health quality of life scale, *LS* least squares, *PEMBRO* pembrolizumab, *IPI + NIVO* ipilimumab and nivolumab, *CI* confidence interval, *VAS* Quality of life visual analog scale, *PEMBRO* pembrolizumab, *IPI + NIVO* ipilimumab and nivolumab

Between treatments, patients receiving PEMBRO demonstrated an adjusted LS mean improvement 3.7 points greater than IPI + NIVO patients (95% CI 1.4, 6.1; *p* = 0.002) in QLQ-C30 GHS/QoL at week 12; 3.2 points greater (95% CI 0.8, 5.6; *p* = 0.008) at week 18 and 3.0 points greater (95% CI 0.8, 5.5; *p* = 0.01) at week 24 vs IPI + NIVO (Fig. 2).

### EQ-VAS LS mean change from baseline

Within-treatment VAS change from baseline was not significantly different by week 24 for either PEMBRO or IPI + NIVO. Between treatments, patients receiving PEMBRO demonstrated an adjusted LS mean improvement in EQ-VAS 1.5 points greater than IPI + NIVO patients (95% CI − 0.5, 3.9; *p* = 0.1) at week 12; 1.9 points greater (95% CI -0.01, 3.9 *p* = 0.06) at week 18; and 2.6 points greater (95% CI 0.6, 4.5; *p* = 0.01) at week 24 vs IPI + NIVO.

### Patients having therapy objective response

**Fig. 3:**
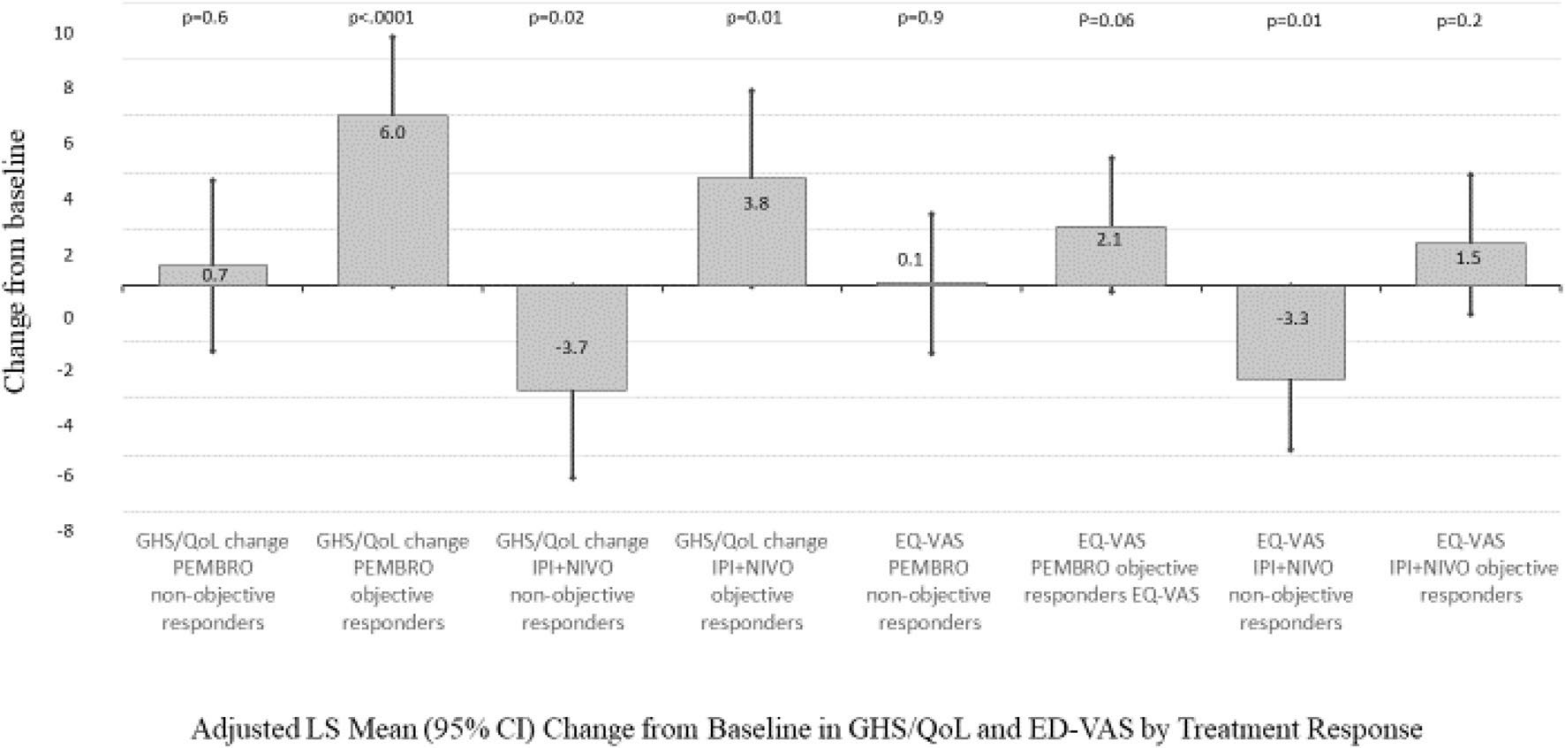
Adjusted LS Mean (95% CI) Change from Baseline in GHS/QoL and ED-VAS by Treatment Response. Repeated measures mixed model with cohort by objective response interaction, cohort by week interaction controlling for baseline EQ-VAS comorbidity index, ECOG at baseline, age, AJCC stage, autoimmune comorbidity, therapy objective response to therapy. *AJCC* American Joint Committee on Cancer, *ECOG* Eastern Cooperative Oncology Group, *CI* confidence interval, *GHS/QoL* global health quality of life scale, LS = Least squares, *PEMBRO* pembrolizumab, *IPI + NIVO* ipilimumab and nivolumab, *CI* confidence interval, *VAS* quality of life visual analog scale, *PEMBRO* pembrolizumab, *IPI + NIVO* ipilimumab and nivolumab

In patients having a therapy objective response, those treated within the PEMBRO group had a statistically significant increase in QLQ-C30 GHS/QoL of 6.0 points (95% CI 3.1, 8.8; *p* < 0.0001) and those treated with IPI + NIVO reported a statistically significant 3.8 point increase (95% CI 0.8, 6.9; *p* = 0.01). PEMBRO patients not achieving a therapy objective response reported no change in QLQ-C30 GHS/QoL at week 24 (0.7, 95% CI − 2.3, 3.7; (*p* = 0.6), while IPI + NIVO non-responders deteriorated significantly, − 3.7 points (95% CI − 6.8, − 0.6; *p* = 0.02). IPI + NIVO was associated with a significant within-group decrease in EQ-VAS in patients not achieving a therapy objective response (− 3.3, 95% CI − 5.8, − 0.8; *p* = 0.01). PEMBRO patients’ maintained baseline QoL, irrespective of treatment response (Fig. 3).

### Clinically meaningful change from baseline, EORTC QLQ-C30 GHS/QoL, EQ-VAS

**Fig. 4:**
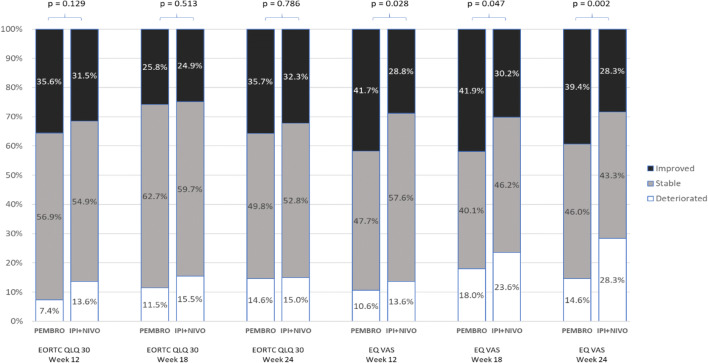
Clinically meaningful change in EORTC QLQ-C30 GHS and EQ-VAS scores. *GHS/QoL* global health quality of life scale, *PEMBRO* pembrolizumab, *IPI + NIVO* ipilimumab and nivolumab, *EQ VAS* quality of life visual analog scale

At week 24, 39.4% of PEMBRO patients demonstrated clinically meaningful improvements from baseline in EQ-VAS compared with 28.3% of IPI + NIVO patients (*p* = 0.002). (Fig. 4). However, there were no differences in proportion of patients reaching the definition of clinically meaningful change from baseline to week 24 in EORTC QLQ-C30 GHS/QoL.

### Functional scales and symptom scores

**Fig. 5:**
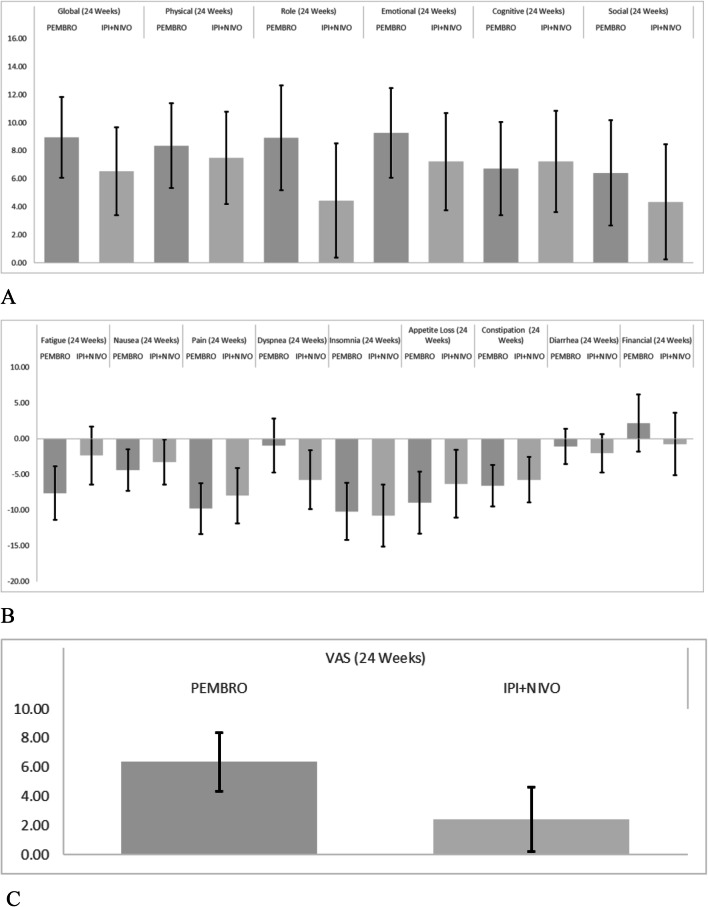
Unadjusted Mean (95% CI) Change from Baseline for EORTC QLQ-30 Functional Scales, Global Health Status/QoL (**a**) and Symptom Scores (**b**), and EQ-VAS (**c**) at Week 24. For global health status/quality of life score and all functional scales, a higher score denotes better quality of life or function. For symptom scores, a lower score denotes fewer symptoms. For the EQ-VAS, higher score denotes better quality of life. *VAS* Visual analog scale, *PEMBRO* pembrolizumab, *IPI + NIVO* ipilimumab + nivolumab,

Change in functional scores derived from the EORTC QLQ-C30 are presented descriptively in Fig. 5a and symptom scores are presented descriptively in Fig. 5b. Unadjusted for differences in baseline characteristics, the largest change observed in functioning scores were in emotional functioning with PEMBRO-treated patients increasing 9.2 (95% CI 7.2, 12.6), and IPI + NIVO patients increasing 7.2 (95% CI 3.9, 10.6). Role functioning improvements were 8.9 (95% CI 4.4, 12.7) for PEMBRO, and 4.4 (95% CI 0.4, 8.5) for IPI + NIVO. The largest symptom reductions were in pain scores with PEMBRO demonstrating a 9.7 (95% CI 5.9, -13.6) reduction, and IPI + NIVO having a 8.0 (95% CI 4.4, 11.5) reduction. Insomnia was reduced 10.2 [95% CI 6.2, 14.2) in PEMBRO patients and 10.7 [95% CI 6.4, 15.1) with IPI + NIVO.

## Discussion

This study examined QoL in real-world patients. Real-world studies do not exclude those who may not qualify for clinical trials due to factors influencing QoL such as poorer performance status and significant comorbidity [4, 5]. In contrast to clinical trials, we collected longitudinal data on enrolled patients through 24 weeks after initiating therapy without removing those who fail to achieve an objective treatment response. In our study, PEMBRO was associated with greater improvement in scores indicating better QoL from baseline to week 24 for both the QLQ-C30 GHS/QoL and EQ-VAS, compared to IPI + NIVO. Although statistically significant, the mean differences in QoL for either instrument did not meet the criteria for clinically significant superiority. These results align with KEYNOTE-006 (PEMBRO vs IPI), which demonstrated a QLQ-C30 GHS/QoL score decrease of − 10.0 for IPI vs a more modest score decrease of − 1.9 and − 2.5 for the two PEMBRO treatment arms; *p* < 0.001.

In the real-world patients studied, we found that baseline mean QLQ-C30 GHS/QoL scores were lower than observed in clinical trials [4, 5]. In the PEMBRO arm, baseline means (SD) QLQ-C30 GHS/QoL was 58.8 (22.3) and 59.5 (22.6) for the IPI + NIVO arm. This contrasts with baseline mean (SD) QLQ-C30 GHS/QoL scores in KEYNOTE-006 for PEMBRO of 71.4 (20.4); 70.5 (21.9); and IPI: 67.4 (24.0). In CHECKMATE-067, baseline mean (SD) QLQ-C30 GHS/QoL for NIVO was 74.7 (19.4) and 70.7 (22.3) for IPI + NIVO. In CHECKMATE-069, NIVO + IPI and IPI had similar mean baseline QLQ-C30 GHS/QoL scores (76.9 vs 80.9), respectively. The findings may be related to differences between real-world and trial populations, as these trials exclude poor performance status subjects.

In our study, if patients responded to treatment, both treatments had improved QoL at week 24 compared to baseline, slightly greater but not statistically significant with PEMBRO. However, there was statistically significant worsening of QoL in IPI + NIVO vs PEMBRO patients not achieving an objective therapy response according to both the QLQ-C30 GHS/QoL and EQ-VAS. PEMBRO non-responders did not worsen. It is possible that when controlling for baseline clinical differences in treatments in the real-world in those not achieving response to therapy, QoL may be negatively influenced by the impact of differing risk–benefit profiles of the treatments. Interpreting QoL in cancer patients receiving treatment is challenging as it is difficult to separate the impact of QoL from the treatment versus the disease, given relevant patient characteristics. When we analyzed the QoL of the two groups of patients overall controlling for disease and patient characteristics that differed significantly between the cohorts, we did not see a large difference in QoL. However, when we specifically focused on patients who were not benefiting from therapy, we found that the IPI + NIVO group’s QoL declined more than the PEMBRO group suggesting that the cause of the decline was at least partially related to the treatment. While some of the decline in QoL could be attributed to differences in patient characteristics, we believe this is less likely given that the PEMBRO group had worse baseline features. However, clinical relevance of a two-year difference in mean age, and a 0.4 difference in comorbidity index is not known.

The results of this study highlight that QoL is a multidimensional construct, and that a single summary score complements the information provided through dimension specific QoL scores. If treatments have different AEs, the differential impact on various aspects of QoL are potentially captured by QoL measures with dimension specific scores. Global QoL ratings or summary scores reveal the overall impact, either through explicit or implicit weighting of the various dimensions of health relevant to an individual. For these reasons, rather than view the issue as a matter of accuracy or bias, the results demonstrate the importance of reporting each relevant outcome that differentiates the impact of treatment—AEs, dimension-specific impact, and overall QoL impact. If all of these outcomes favor a specific treatment, the more robust the results. Otherwise, it is important to note that how the treatments have differential impact on specific aspects of QoL potentially due to clinical heterogeneity of response to treatment, and/or different preferences for various aspects of health. CHECKMATE-069 reported treatment-related grade 3–4 adverse events in 54% of patients receiving IPI + NIVO compared with 20% of patients who received IPI alone. In this study, however, treatment arms similarly maintained QLQ-C30 GHS/QoL at week 7 (69.2 vs 74.5) and at week 13 (78.5 vs 72.2), IPI + NIVO and IPI, respectively, despite differences in AEs [10]. CHECKMATE-067 also reported that despite differences in the rate of grade 3–4 adverse events (IPI + NIVO [58.5%], NIVO [20.8%], and IPI [27.7%]) [6], this did not result in differences in QoL. Neither CHECKMATE-067 or CHECKMATE-069 examined objective responder subgroups, however.

QoL as an outcome has been gaining importance within clinical trials to understand the patient’s perspective [15]. A recent systematic review of 49 oncology treatments seeking 64 indications indicated that 70.3% included PRO data in their regulatory submissions [16]. Many recent clinical trials in AM immunotherapies have specifically collected QoL data [4, 5, 17–23]. To-date, there is a dearth of published real-world PRO evidence, with only one small cross sectional study identified, assessing a small sample (*n* = 41) at one timepoint in the course of their therapy, either just prior to IPI initiation, or at an interval thereafter [24]. Our study is one of the first to generate longitudinal evidence on the impact of immuno-oncology treatments on QoL in AM treatment in the real-world.

An unusual feature of this study is the extremely high rate of retention, completion and compliance (> 90%). This is due in part to the study design, in that patients were not excluded from long term follow-up if progressing on therapy. Also in recent trials, patients were required to complete PRO instruments in the clinic prior to the study visit so that PRO responses would not be influenced by information regarding response to therapy during the visit [5]. No attempt to encourage subjects to complete instruments was made after the study visit if the PRO could not be completed before the consultation. In our study, patients were given a 10-day window around measurement timepoints with reminders in order to complete instruments, enhancing completion and compliance. It is also possible that treating clinicians introduced selection bias in enrolling patients they believed would be most likely to complete study measurements.

This study has several limitations. Notably, real-world treatment comparisons are potentially subject to selection bias, as random allocation of patients to treatments cannot be employed to achieve balanced characteristics across treatment arms. The PEMBRO cohort was older and had more comorbidity, than IPI + NIVO patients, which may have impacted QoL. As sites did not capture information about number and characteristics of eligible patients that declined to participate in the study, generalizability is limited. Data collection of clinical characteristics and objective treatment response relied on retrospective medical chart review. As such, results are limited by the completeness of information that was recorded in those charts and data that was collected. Study parameters important in potentially impacting QoL such as ECOG performance status are not always assessed or recorded in routine medical practice. However, as an assessment of frailty can be made using clinical judgement and patient objective presentation during a clinical consultation, this may or may not be a limitation in extrapolating our findings to routine practice. Baseline assessment of HRQL was not true baseline as there may have been a delay between questionnaire completion and initiation of treatment; the questionnaire was mailed within up to ten days of starting therapy, not completed at initiation of treatment. This study did not capture the occurrence of adverse events, as these events may be unreliably captured outside of a clinical trial setting. Our study only examined QoL associated with PEMBRO as a single agent PD-1 inhibitor, although nivolumab was also approved for monotherapy in this indication; as such, no real-world data are available on QoL for patients treated with nivolumab as a monotherapy. This was a real-world observational study of patients whose treating clinician had made the decision to initiate either PEMBRO or IPI + NIVO. We did not capture the clinician’s reasoning for choosing one treatment over the other and did not intervene in treatment selection. As such, we expected patient specific characteristics may have impacted treatment selection Although we captured QoL improvement, deterioration or stability at each measurement point, we did not attempt to correlate that with treatment response.

In conclusion, these exploratory PRO analyses from the real-world indicate that QoL was better maintained with PEMBRO than with IPI + NIVO when administered as first line therapy in patients with advanced melanoma. Further real-world studies are warranted to replicate our findings. Combined with the understanding of PFS, OS and toxicity risk with each treatment derived from trial data, this data can support treatment decision making.

## Electronic supplementary material

Below is the link to the electronic supplementary material.

Supplementary file1 (DOCX 15 kb)

## Footnotes

1 Alopecia areata, Autoimmune hemolytic anemia, Autoimmune hepatitis, Dermatomyositis, Diabetes (type 1), Autoimmune juvenile idiopathic arthritis, Glomerulonephritis, Graves’ disease, Guillain-Barré syndrome, Idiopathic thrombocytopenic purpura, Myasthenia gravis, Autoimmune myocarditis, Multiple sclerosis, Pemphigus/pemphigoid, Pernicious anemia, Polyarteritis nodosa, Polymyositis, Primary biliary cirrhosis, Psoriasis, Rheumatoid arthritis, Scleroderma/systemic sclerosis, Sjögren’s syndrome, Systemic lupus erythematosus, Hashimoto’s thyroiditis, Autoimmune of uveitis, Vitiligo, Granulomatosis with polyangiitis (Wegener’s).
