# Supplementary material for: Health-related quality of life (QoL) in patients with advanced melanoma receiving immunotherapies in real-world clinical practice settings
Source: Qual Life Res. 2020 May 13;29(10):2651–60. doi: 10.1007/s11136-020-02520-7 (PMC7561540; doi:10.1007/s11136-020-02520-7)
Supplement: Supplementary file 1 — Supplementary file1 (DOCX 15 kb) [file 11136_2020_2520_MOESM1_ESM.docx]

Supplemental Table 1: Completion and Compliance Rates EORTC QLQ-C30 and EQ-5D-5L*

|  |  | PEMBRO  (N =225) | IPI + NIVO  (N =187) |
| --- | --- | --- | --- |
| Baseline | Expected to Complete Questionnaires | 225 (100) | 187 (100) |
|  | Completion | 225 (100) | 187 (100) |
|  | Compliance | 225 (100) | 187 (100) |
| Week 6 | Expected to Complete Questionnaires | 225 (100) | 186 (99.5) |
|  | Completion | 222 (98.7) | 186 (99.4) |
|  | Compliance | 222 (100) | 186 (100) |
| Week 12 | Expected to Complete Questionnaires | 223 (99.1) | 186 (99.5) |
|  | Completion | 220 (97.8) | 186 (99.5) |
|  | Compliance | 220 (98.7) | 186 (100) |
| Week 18 | Expected to Complete Questionnaires | 220 (97.8) | 183 (97.9) |
|  | Completion | 217 (96.4) | 181 (96.8) |
|  | Compliance | 216 (98.6) | 181 (98.9) |
| Week 24 | Expected to Complete Questionnaires | 216 (96.0) | 180 (96.3) |
|  | Completion | 213 (94.6) | 180 (96.3) |
|  | Compliance | 213 (98.6) | 180 (100) |

*Compliance Rate, defined as the percentage of PRO measurements over number of eligible subjects who are expected to complete the PRO assessment, excluding those missing by death. Completion Rate defined as the percentage of PRO measurements over all patients enrolled at baseline, irrespective of death or loss to follow-up. PEMBRO = Pembrolizumab, IPI+NIVO = Ipilimumab + nivolumab
